# Dysregulation of Ephrin receptor and PPAR signaling pathways in neural progenitor cells infected by Zika virus

**DOI:** 10.1080/22221751.2020.1818631

**Published:** 2020-09-20

**Authors:** Sathya N. Thulasi Raman, Elyse Latreille, Jun Gao, Wanyue Zhang, Jianguo Wu, Marsha S. Russell, Lisa Walrond, Terry Cyr, Jessie R. Lavoie, David Safronetz, Jingxin Cao, Simon Sauve, Aaron Farnsworth, Wangxue Chen, Pei-Yong Shi, Youchun Wang, Lisheng Wang, Michael Rosu-Myles, Xuguang Li

**Affiliations:** a Centre for Biologics Evaluation, Biologics and Radiopharmaceutical Drugs Directorate, HPFB, Health Canada and WHO Collaborating Centre for Standardization and Evaluation of Biologicals, Ottawa, ON, Canada; b National Microbiology Laboratory, Public Health Agency of Canada, Winnipeg, MB, Canada; c National Research Council of Canada, Human Health Therapeutics, Ottawa, ON, Canada; d Department of Biochemistry & Molecular Biology, University of Texas Medical Branch, Galveston, TX, USA; e National Institute for Food and Drug Control and WHO Collaborating Center for Standardization and Evaluation of Biologicals, Beijing, People’s Republic of China; f Department of Biochemistry, Microbiology and Immunology, Faculty of Medicine, University of Ottawa, Ottawa, ON, Canada

**Keywords:** Neural progenitor cells, ZIKV, Ephrin signaling, PPAR signaling, proteomics, ingenuity pathway analysis

## Abstract

Zika virus (ZIKV) infection is a serious public threat with cases reported in about 70 countries and territories. One of the most serious consequences of ZIKV infection is congenital microcephaly in babies. Congenital microcephaly has been suggested to result from infection of neural progenitor cells (NPCs) in the developing fetal brain. However, the molecular and cellular mechanisms underlying microcephaly development remains to be fully elucidated. In this study, we employed quantitative proteomics to determine protein expression profile that occur during viral replication in NPCs. Bioinformatics analysis of the protein expression changes resulted in the identification of a wide range of cell signaling pathways. Specifically, pathways involved in neurogenesis and embryonic development were markedly altered, along with those associated with cell cycle, apoptosis, lipid metabolism and oxidative stress. Notably, the differential regulation of Ephrin Receptor and PPAR signaling pathways, as revealed by quantitative proteomics and validated by qPCR array, underscores the need to explore these pathways in disease development. Collectively, these results indicate that ZIKV-induced pathogenesis involves complex virus-host reactions; the findings reported here could help shed light on the mechanisms underlying ZIKV-induced microcephaly and ZIKV replication in NPCs.

## Introduction

Zika virus (ZIKV) is an arthropod-borne (arbovirus) pathogen of the *flaviviridae* family, which includes other human pathogens of importance such as dengue virus, chikungunya virus, yellow fever virus and Japanese Encephalitis virus. Vectors from the Aedes genus, such as Aedes aegypti and Aedes albopictus, transmit the virus. ZIKV is a significant pathogen due to its ability to cause a wide spectrum of neurological complications [1]. ZIKV came into the limelight during the Brazilian 2015–2016 outbreak, when widespread congenital infections resulted in severe birth defects in infants born to ZIKV-infected pregnant mothers [1]. In 2018, another outbreak was reported in India, where 190 cases of ZIKV infection were confirmed [2]. A recent long-term study on healthy babies born to ZIKV-infected pregnant mothers concluded that these babies, who did not have any clinical evidence of congenital ZIKV syndrome at birth, were at a higher risk of developing neurocognitive deficits during the first 18 months of life [3]. While the most prominent and striking effects of ZIKV infections have been observed in infants, adults can also suffer from neurological diseases such as GBS, acute myelitis, encephalomyelitis, encephalitis, meningoencephalitis, and sensory polyneuropathy [1].

Several studies have shown that ZIKV preferentially targets the neural progenitor cells (NPCs), which are the precursor cells of a variety of neuronal cell types in the developing fetal brain [4]. These studies showed that ZIKV infection leads to cell-cycle arrest, apoptosis and defects in differentiation of NPCs, which in turn could contribute to the development of microcephaly. Additionally, infection of neural stem cells also results in alteration in the expression of neuronal cell type markers [5]. Altogether, these studies emphasize the ability of ZIKV to profoundly alter the differentiation and proliferative capability of infected NPCs. Many studies have analyzed the changes in the proteomic and transcriptomic landscape of the host during infection [6, 7]. However, the intricate and complex signaling pathways leading to impaired neurogenesis and the effects of these pathways on ZIKV replication and spread are not fully understood.

In this study, we investigated cellular responses in iPSC (Induced Pluripotent Stem Cell)-derived NPCs infected by ZIKV. iPSCs are derived from adult cells that have been reprogrammed to an embryonic stem cell-like state by forced expression of certain genes [8]. Their pluripotent nature make them amenable for differentiation into a multitude of cell types, including NPCs. iPSC-derived NPCs are considered a good model to study ZIKV host interactions and valuable information has been obtained from studies employing these cells in models of ZIKV infection [4, 5, 7]. In this study, we infected iPSC-derived NPCs with ZIKV and investigated ZIKV-induced cellular responses by analyzing the proteome changes using mass spectrometry. We identified several novel ZIKV-induced cell signaling pathways and proteins that have key roles in neurogenesis and viral replication and validated two pathways, “Ephrin receptor signaling” and “PPAR signaling,” through qPCR analysis. In addition, we also confirmed protein-level changes in the expression of some of the upregulated genes identified in the PPAR signaling pathway.

## Materials and methods

### Cell lines, virus and media

C6/36 cells, used for ZIKV virus propagation were maintained in EMEM media (ATCC) with 10% FBS (GIBCO). Vero cells were maintained in DMEM (GIBCO) supplemented with 10% FBS (GIBCO).

iPSC-derived NPCs were kindly provided by Dr. William Stanford of Ottawa Hospital Research Institute, Ottawa, ON. The iPSC cells were generated from skin fibroblasts (Coriell Institute, NJ, USA; Repository ID - GM00969) of a healthy 2 year-old female donor [9]. iPSC-derived NPCs were maintained in NPC media containing 1:1 DMEM-F12 (Thermo Scientific, cat No. 11330-057):Neurobasal media (Thermo Scientific, Cat No. 21103-049) supplemented with 0.5X N2 (Thermo Scientific, Cat No. 17502-048), 0.5X B27 (Thermo Scientific, Cat No. 17504-044), 5 µg/ml Insulin (Wisent, Cat No. 511-016-CM), 0.5X Glutamax (Thermo Scientific, Cat No. 35050-061), 50 µg/ml Gentamicin (Thermo Scientific, Cat No. 15750-060), 20 ng/ml FGF-2 (Fisher Scientific, Cat No. PHG0266) and 20 ng/ml EGF (Sigma Aldrich, Cat No. E9644). The ReNcell CX cells (Millipore Sigma, Cat No. SCC007) were maintained in ReNcell NSC maintenance media (Millipore Sigma, Cat No. SCM005), supplemented with FGF-2 (Fisher Scientific, Cat No. PHG0266) and EGF (Sigma Aldrich, Cat No. E9644) to a final concentration of 20 ng/ml each. The iPSC-derived NPC cells and the ReNcell CX cells were maintained at 37°C in 5% O_2_ and 10% CO_2_.

Detailed information on iPSC cell culture maintenance and differentiation into NPCs is described in the Supplementary Methods.

### Virus stock preparation

C6/36 cells were plated in a 150 mm dish at a density of 1.3 × 10^7^ cells/dish in EMEM media with 10% FBS the day before infection. The next day, for virus adsorption, the cells were incubated with viral inoculum diluted in EMEM at a MOI of 0.01 for 1 h at 37°C with intermittent rocking every 15 min. The viral inoculum was removed after 1 h and the cells were replenished with EMEM media containing 2.5% FBS. The infected cell media was collected upon observation of complete disruption of the monolayer (approximately 4–5 dpi) and centrifuged to remove cellular debris. The supernatant was collected and stored in −80°C after addition of FBS to bring the final concentration to 20%. This frozen supernatant was used as the virus stock for subsequent infection experiments.

### Plaque forming assay

Virus titer in infected cell supernatant was determined by Plaque forming assay on Vero cells. Vero cells were plated at a density of 4 × 10^5^ cells/well in 6-well plates. Next day, serial 10-fold dilutions of the supernatant were prepared in DMEM media and the virus adsorption was allowed to proceed by incubating the cells with the prepared dilutions for 1 h at 37°C with intermittent rocking every 15 min. The infected cell monolayer was then rinsed with PBS and then overlaid with 0.8% Carboxymethyl cellulose (CMC) (SIGMA, Cat No. C5013)/DMEM media supplemented with 2.5% FBS and incubated at 37°C for 4 days. At 4 days post infection, the CMC/DMEM media overlay was removed and the plaques were visualized by staining the monolayer with Crystal violet solution (SIGMA, Cat No. HT90132).

### Immunostaining

For immunostaining, the cells were fixed with 4% Paraformaldehyde in PBS and permeabilized with 0.5% TritonX-100 in PBS. The fixation and permeabilization solutions were prepared in PBS (2.7 mM KCl, 137.93 mM NaCl, 1.763 mM KH_2_PO_4_, 10.143 mM Na_2_HPO_4_, pH 7.4). The differentiation status of the NPCs was then confirmed by immunostaining with antibodies against NPC markers Sox2 (1:100) (EMD Millipore, Cat No. AB5603), Nestin (1:200) (R&D systems, Cat No. MAB1259) and Pax-6 (1:350) (Abcam, Cat No. ab195045). Viral replication was detected by immunostaining with mouse anti-J2 dsRNA (1:120) (Scicons, Cat No.10010200) and rabbit- anti ZIKV envelope (1:200) (Novus Bio, NBP2-52666). The expression of PPAR pathway proteins in infected cells was determined by immunostaining with antibodies against RXRG (1:200) (Abcam, Cat No. ab15518) or HELZ2 (1:100) (Abcam, Cat No. ab129781) or APOC3 (1:100) (Abcam, Cat No. ab55984) or FGR (1:100) (Thermo Scientific, Cat No. 703189) and Zika Envelope protein (1:500) (BioFront Technologies, Cat No. BF-1176-56). The cells were then counterstained with anti-mouse 488 (1:200) (Jackson Immunoresearch, Cat No. 115545146) and anti-rabbit Cy3 (1:200) (Jackson Immunoresearch, Cat No. 111165144). Nuclei were stained with DAPI (1:1000) (SIGMA, Cat No. D9542) and coverslips were mounted onto the slides with Prolong Glass Antifade reagent (Life Technologies, Cat No. P36982).

### ZIKV infection of iPSC-derived NPCs

NPCs were plated at a density of 1.2 × 10^6^ cells/well in 60 mm dishes or at a density of 7.7 × 10^4^ cells/well in 4-well chamber slides in NPC media. The cells were infected the next day at a MOI of 5 with ZIKV. Virus inoculum was prepared by diluting the virus stock in NPC media and virus adsorption was allowed to proceed by incubating the cells with the inoculum for 1.5 h at 37°C with intermittent rocking every 15 min. Mock infected cells were treated similarly with NPC media that contained no virus. After adsorption, the cells were rinsed once with PBS, overlaid with NPC media and then incubated at 37°C for 24, 48 or 72 h.

### Rencell CX infection for immunostaining

Rencell CX cells were plated at a density of 3 × 10^4^ cells/well in 8-well chamber slides (Thermo Fisher Scientific, Cat No. 154534PK) in ReNcell maintenance media containing growth factors (ReNcell complete media). Next day, virus inoculum was prepared by diluting the stock in ReNcell complete media at a MOI of 5 and virus adsorption was allowed to proceed by incubating the cells with the inoculum for 1.5 h at 37°C with intermittent rocking every 15 min. Mock infected cells were treated similarly with NPC media that contained no virus. After adsorption, the cells were rinsed once with PBS, overlaid with ReNcell complete media and then incubated at 37°C for 48 h.

### Proteomics analysis

NPC monolayer was lysed and collected in RIPA buffer. The proteins in the lysate were precipitated using cold acetone and reduced with DTT. The samples were carbamidomethylated and digested using *r*Lys-c and trypsin enzymes. The protein digests were reverse phase separated and eluting peptides were analyzed with Orbitrap^TM^ Fusion^TM^ Tribrid^TM^ mass spectrometer (Thermo Fisher Scientific). MS/MS spectra were processed using Proteome discoverer.

Detailed information on methodology used for sample preparation and the proteomic analysis itself is described in Supplementary Methods.

### Quantitative PCR array

The RNA from ZIKV-infected NPCs was extracted using the RNeasy Plus Mini Kit (Qiagen), as per the manufacturer’s instructions. RNA (1.5–2 µg) was reverse transcribed using Superscript IV VILO (Invitrogen) and 15–20 ng of cDNA was used per reaction for quantitative PCR using Taqman Fast Advanced Mastermix (Applied Biosystems). Amplification of the PCR products was carried out using Applied Biosystems 7500 real-time PCR system. Fold change of target genes in infected cells compared to mock was calculated using the 2^-ΔΔCt^ method of relative quantitation, where Ct values of the housekeeping gene GAPDH was used for normalization [10]. The qPCR data was generated with three biological replicates and at least two independent experiments.

Custom Taqman array FAST 96 well plates for assessing mRNA expression of genes belonging to the Ephrin signaling pathway and the PPAR signaling pathway were purchased from Thermofisher Scientific.

## Results

### ZIKV productively infects iPSC-derived NPCs

**Figure 1.**
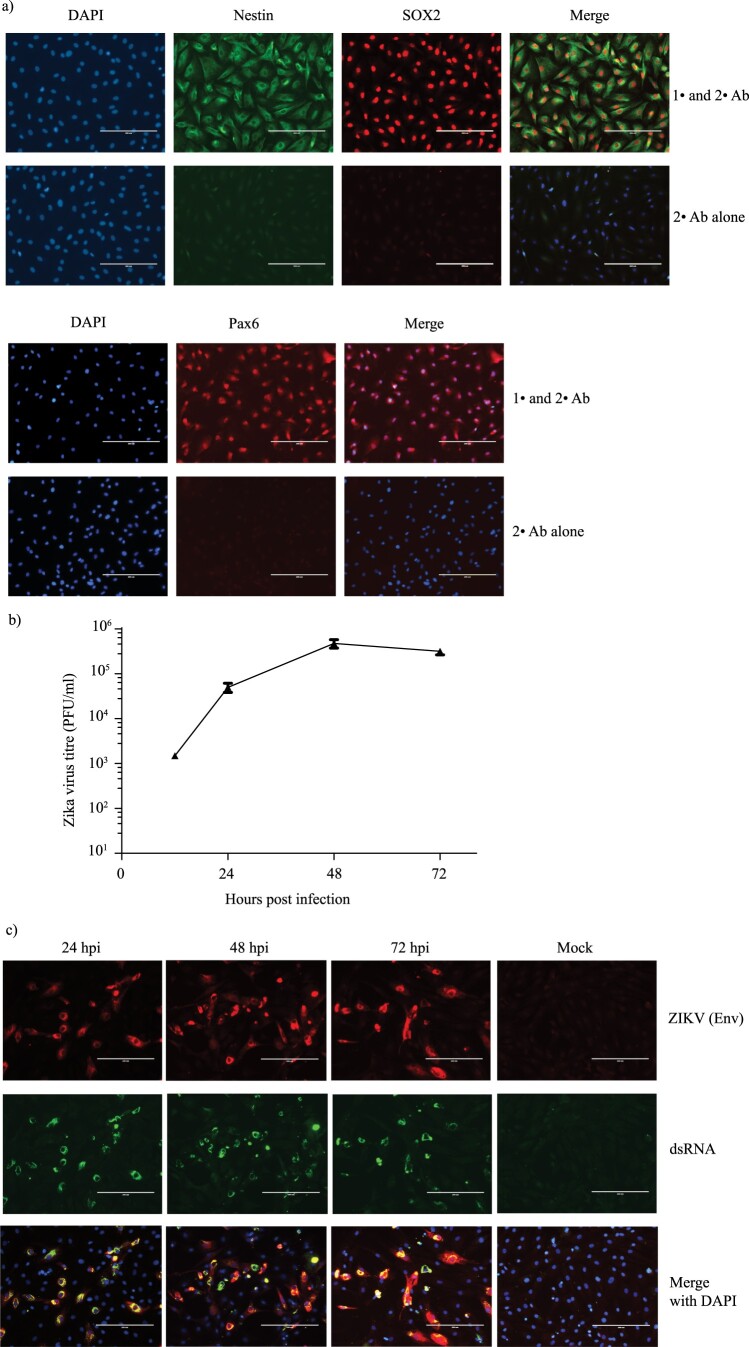
ZIKV productively infects iPSC-derived NPCs. (a) NPCs were immunostained with primary (1°) antibodies against NPC markers SOX2 (red) and Nestin (green) (Top panel) or Pax6 (red) (bottom panel). As a negative control, NPC cells were immunostained with secondary (2°) antibodies alone. The nuclei were visualized using DAPI (blue). Scale bar is 200 µm. (b) NPC cells were infected with ZIKV at a MOI of 5 in triplicates and cell culture supernatant collected at 12 h, 24 h, 48 h and 72 hpi. Mean virus titers determined by plaque forming assay and the corresponding standard deviations as error bars are plotted in the graph. (c) Virus replication in ZIKV-infected NPCs was monitored by staining the infected cells at various time points with antibodies against the virus envelope protein (red) and double stranded (ds) RNA (green). Nuclei were visualized using DAPI (blue). Scale bar is 200 µm.

The effects of Zika virus infection on NPCs in the developing fetal brain is believed to be the primary cause of microcephaly in infants born to ZIKV-infected mothers [4]. To verify that our iPSC-derived NPCs were an appropriate model, we first tested their capacity to support productive ZIKV infection. We confirmed the identity of our NPC cells by immunostaining with NPC specific markers Nestin, SOX2 and Pax6 [11] (Figure 1(a)). The cells were infected with the American strain PRVABC59 [12] and the production of infectious virus at various time points was determined using plaque assay (Figure 1(b)). As shown in the figure, ZIKV titers steadily increased from 12 hrs until 48 hrs post infection (hpi), before starting to plateau at 72 hrs. Additionally, we also confirmed the expression of viral Envelope protein and the presence of dsRNA in infected cells, which serves as a marker for replication of RNA viruses [13] (Figure 1(c)).

### ZIKV infection alters expression of multiple proteins in NPCs

**Figure 2.**
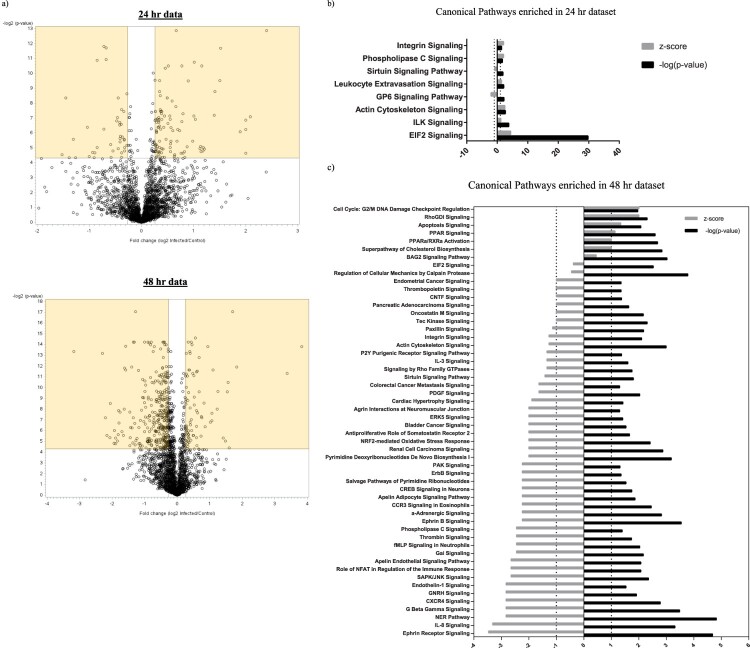
Volcano plot and canonical pathways identified by IPA using the protein expression dataset. (a) Fold change protein expression and the associated *p*-values of the 24 and 48 hpi dataset were plotted to obtain a Volcano plot. Proteins with significant *p*-values and with a fold change value ≥+1.2 or ≤−1.2 are highlighted. (b & c) IPA was used to identify the Canonical cellular pathways represented by the differentially expressed proteins in ZIKV-infected cell lysates collected at 24 and 48 hpi. The data from IPA Canonical pathway analysis were used to plot a graph of the activation state (z-score) and significance (-log(p) value) of the pathways represented by the (b) 24 hpi dataset and (c) 48 hpi dataset using GraphPad Prism (version 7). Pathways that are activated have a positive z-score and repressed pathways have a negative z-score. The Threshold of z<−1 and z>1 that we set for selection of activated and repressed pathways for further analysis is displayed as a dotted line in the graph.

Having confirmed the ability of primary NPCs to support productive ZIKV infection, we employed mass spectrometry and proteomic analysis to decipher and quantitate differentially regulated cellular proteins during virus infection. Since the peak titers were observed at 48 hpi (Figure 1(b)), we collected the cell lysate at 48 hpi and at an earlier time point of 24 hpi for proteomic analysis. A total of 2276 proteins were identified in the cell lysate collected at 24 hpi and 2239 proteins were identified in the cell lysate collected at 48 hpi with 95% or higher confidence at the peptide and protein levels (Table S1). The fold change threshold for differentially expressed cellular proteins was set as ≥+1.2-fold or ≤−1.2 fold compared to expression in mock-infected group and the significance of the differential expression was determined by statistical tests. Using these criteria, we identified 102 proteins to be upregulated and 40 proteins to be downregulated at 24 hpi, while 72 proteins were upregulated and 324 proteins were downregulated at 48 hpi (Table 1 and Table S2), which are represented in the upper right-hand and upper left-hand quadrant (highlighted) of the volcano plot respectively (Figure 2(a)).

**Table 1.** Top differentially regulated host proteins affected by infection at 24 and 48 hpi.

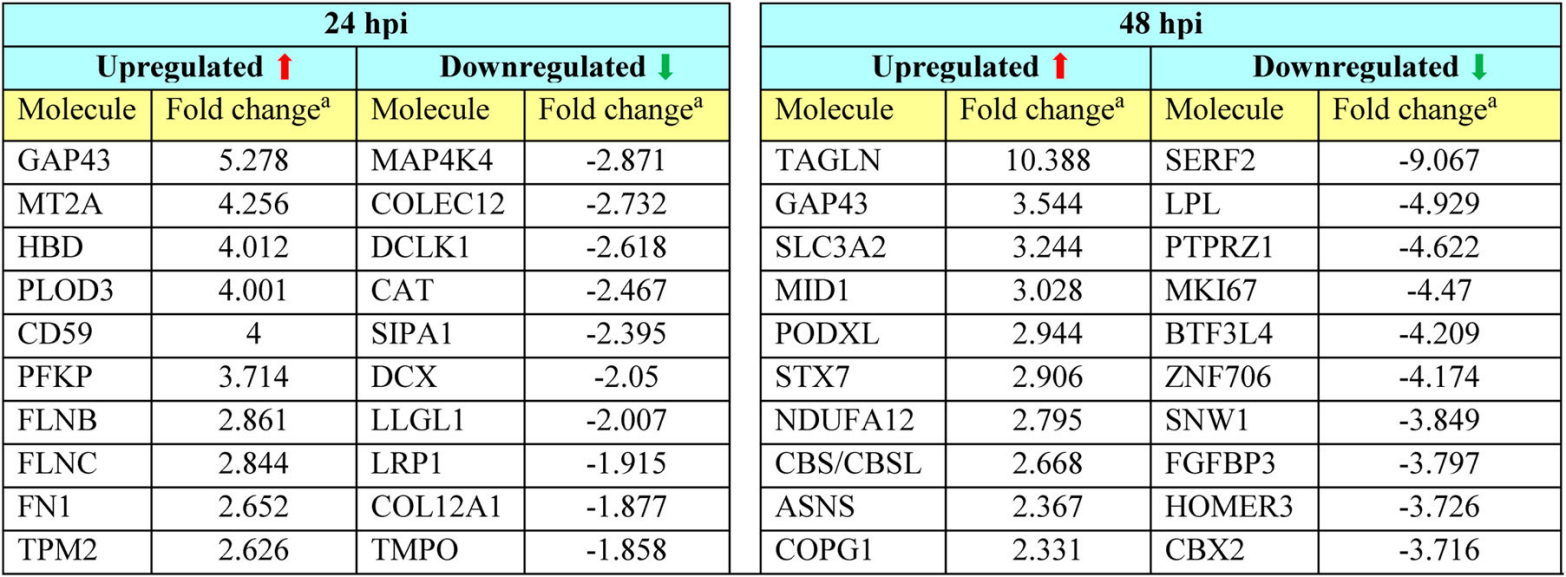

^a^Fold change in expression in infected cell lysate when compared to mock infected cell lysate.

Gene ontology analysis of the top 10 differentially expressed (top 10 upregulated and top 10 downregulated) proteins at 24 hpi (Table 1) revealed enrichment for biological processes such as cell differentiation, cell development, neuron projection development and axonogenesis (Table 2). This is noteworthy, as dysregulation in cell differentiation, neurogenesis and cell death pathways are believed to be the primary mechanisms leading to the development of ZIKV-induced microcephaly [4]. Several of the top differentially regulated proteins identified at 24 hpi and 48 hpi in our study have previously been known to be differentially expressed during ZIKV or other flavivirus infections. Proteins previously identified to be differentially expressed during ZIKV infection include GAP43, DCX, PTPRZ1, ASNS, SLC3A2 and MKI67 [14–16]. Proteins differentially expressed in flavivirus infections other than Zika include SLC3A2, PODXL, ASNS and LPL [17–19]. Interestingly, mature ZIKV-derived miRNAs target and downregulate CBX2, which was identified to be downregulated in our proteomic analysis [20]. Identification of known proteins that are involved in Zika and other flavivirus infections lends further confidence in our dataset.

**Table 2.** GO enrichment analysis of top differentially expressed proteins at 24 hpi.

GO biological process complete	Reference list^a^	Fold enrichment	Raw *p*-value^b^	FDR^c^
Cell morphogenesis involved in differentiation (GO:0000904)	562	16.7	1.04E-09	8.25E-06
Cell morphogenesis (GO:0000902)	721	14.46	3.51E-10	5.60E-06
Axonogenesis (GO:0007409)	378	13.79	2.50E-05	3.32E-02
Neuron projection morphogenesis (GO:0048812)	490	12.77	5.10E-06	1.16E-02
Plasma membrane bounded cell projection Morphogenesis (GO:0120039)	494	12.66	5.34E-06	1.06E-02
Axon development (GO:0061564)	412	12.65	3.76E-05	4.00E-02
Cellular component morphogenesis (GO:0032989)	825	12.64	1.28E-09	6.79E-06
Cell projection morphogenesis (GO:0048858)	498	12.56	5.59E-06	9.90E-03
Cell part morphogenesis (GO:0032990)	520	12.03	7.14E-06	1.14E-02
Cell morphogenesis involved in neuron differentiation (GO:0048667)	442	11.79	5.24E-05	4.91E-02
Neuron projection development (GO:0031175)	679	9.21	3.20E-05	3.92E-02
Cell development (GO:0048468)	1623	7.07	5.67E-08	2.26E-04
Anatomical structure morphogenesis (GO:0009653)	2177	4.79	1.07E-05	1.55E-02
Cell differentiation (GO:0030154)	3729	3.35	3.25E-05	3.70E-02
Cellular developmental process (GO:0048869)	3822	3.27	4.20E-05	4.18E-02
Cellular component organization (GO:0016043)	5620	3.15	1.01E-07	3.21E-04
Cellular component organization or biogenesis (GO:0071840)	5810	3.05	1.71E-07	4.54E-04

^a^Reference gene list used for the analysis is from Homo sapiens.

^b^*P*-value was determined by Fisher’s Exact test.

^c^FDR is False Discovery rate.

### ZIKV-induced differential expression of cellular proteins results in dysregulation of cell signaling pathways and cellular functions

In order to gain more insight into the effect of these alterations on cellular processes, we analyzed the differentially expressed proteins using a bioinformatics program called Ingenuity Pathway Analysis (IPA). IPA analysis of our proteomics dataset predicted several cellular pathways to be either activated or inhibited upon ZIKV infection (Figure 2(b and c)). While IPA predicted eight signaling pathways to be dysregulated at 24 hpi (Figure 2(b)), the number of pathways predicted to be dysregulated at 48 hpi jumped to 54 (Figure 2(c) and Table S3). At 24 hpi, the most significant and highly regulated pathway was EIF2 signaling. In the 48 hpi dataset, we observed marked alterations of three important pathways critically important in cell cycle regulation, that are “Nucleotide Excision Repair (NER),” “Cell cycle G2/M DNA damage checkpoint regulation” and “Cell cycle control of chromosomal replication” pathways. While, the NER pathway was predicted to be inhibited (z score of −2.828) in ZIKV-infected cells, the Cell cycle G2/M DNA damage checkpoint regulation pathway was predicted to be activated (z score of +2). However, IPA could not assign an activity pattern with confidence for “Cell cycle control of chromosomal replication” pathway (Table S3).

Notably, our analysis also led to the identification of a variety of signaling pathways that could affect virus infection and replication. G protein beta gamma signaling pathway is known to have broad-reaching effects in the host cell. The G proteins α, β and γ are known transducers of the largest family of plasma membrane receptor molecules in eukaryotes called the G protein-coupled receptors (GPCR) [21]. These receptors modulate a wide variety of cellular functions and play an important role in virus replication, which is evident from the existence of a wide variety of host GPCR mimics encoded by viruses. Herpes viruses such as KSHV, HCMV and EBV encode single or multiple viral GPCRs to modulate host cell signaling pathways in order to favour virus replication and dissemination [22]. Additionally, GPCRs are known to affect cellular entry of several viruses such as Marburg and Ebola viruses [23]. In our study, multiple G protein subunits and proteins involved in GPCR signaling were identified to be differentially expressed in ZIKV-infected cells at 48 hpi, which led to the annotation of several cellular pathways that involve cross talk with a GPCR-mediated signaling network. These include Ephrin receptor signaling, SAPK/JNK signaling, Tec Kinase signaling, IL-1 signaling, RhoGDI signaling and Phospholipase C signaling among many others (Table S3 and Figure 2(c)). Other pathways, where the signaling receptor itself is a GPCR include, IL8 signaling, α-Adrenergic signaling, CXCR4 signaling, CCR3 signaling, Apelin Receptor signaling, fMLP signaling neutrophils, GnRH signaling, Thrombin signaling, Antiproliferative Role of Somatostatin Receptor 2, Endothelin-1 signaling. The GPCR signaling receptors involved in the above-mentioned signaling pathways are listed in Table S4. We also found significant alterations in oxidative stress and apoptotic signaling, both of which are dynamic pathways with a profound impact on virus replication and spread [24, 25]. While “NrF2 mediated oxidative stress signaling,” which is a well-studied pathway in virus life cycle [25], was predicted to be repressed, “Apoptosis signaling” pathway was activated at 48 hpi (Figure 2(c) and Table S3).

The impact of ZIKV infection on lipid metabolism was also revealed. As shown in Table S3 and Figure 2(c), cellular pathways related to cholesterol biosynthesis and lipid metabolism such as “Superpathway of Cholesterol biosynthesis” and “PPAR signaling” were predicted to be upregulated in our 48 hpi dataset providing potential novel targets that could be studied for their proviral or antiviral activity. ZIKV, like other flaviviruses, could hijack and upregulate host fatty acid and cholesterol biosynthesis machinery to induce formation of viral replication sites and for various anabolic processes [26]. Appearance of such flavivirus replication sites are intricately associated with significant alterations in lipid metabolism pathways by the virus [26]. Interestingly, innate immune signaling pathways, such as IFN signaling, modulate fatty acid synthesis to create a hostile environment for virus infection and replication [27]. Thus, alterations in lipid metabolic pathways could have a profound effect on virus replication and spread.

Clearly, one of the most serious outcomes of ZIKV infection in pregnant mothers is the congenital abnormalities such as microcephaly; a birth defect where the baby’s head is smaller than expected, with a range of accompanying neurological deficits [4]. To understand whether the altered proteomics profile is associated with biological functions or diseases related to microcephaly, we utilized IPA core analysis. The analysis predicted that the altered protein profile in infected cells resembles the proteomic profile associated with diseases and functions that affect neuronal function such as neurological disease, cellular assembly and organization, protein synthesis, cell death and survival, gene expression, embryonic development and nervous system development (Table 3 and Table S5).

**Table 3.** Top diseases and bio functions affected in infected cells.

Diseases and disorders (24 hpi)		Diseases and disorders (48 hpi)	
Name	*p*-Value range^a^	Name	*p*-Value range^a^
Cancer	4.8E-3–1.94E-12	Neurological disease	3.77E-4–5.08E-13
Organismal injury and abnormalities	4.8E-3–1.94E-12	Organismal injury and abnormalities	4.3E-4–5.08E-13
Tumor morphology	3.16E-3–1.94E-12	Psychological disorders	2.7E-4–5.08E-13
Endocrine system disorders	1.4E-3–1.33E-9	Skeletal and muscular disorders	3.15E-4–1.03E-12
Infectious diseases	1.3E-3–1.57E-7	Infectious diseases	3.77E-4–2.42E-11
Molecular and cellular functions (24 hpi)		Molecular and cellular functions (48 hpi)	
Name	*p*-Value range^a^	Name	*p*-Value range^a^
Protein synthesis	1.3E-3–4.79E-36	RNA post-transcriptional modification	1.04E-5–2.92E-26
RNA damage and repair	2.18E-34–2.18E-34	Cell death and survival	4.1E-4–1.46E-20
Cell death and survival	1.87E-3–1.94E-12	Gene expression	3.14E-4–8.39E-13
Gene expression	4.11E-4–7.09E-10	Cellular assembly and organization	3.65E-4–1.03E-12
Cellular development	3.69E-3–2.64E-09	Cellular function and maintenance	3.77E-4–1.03E-12
Physiological system development and function (24 hpi)		Physiological system development and function (48 hpi)	
Name	*p*-Value range^a^	Name	*p*-Value range^a^
Embryonic development	3.69E-3–9.02E-7	Organismal survival	4.53E-12–4.19E-12
Connective tissue development	3.55E-3–1.78E-6	Nervous system development	3.65E-4–4.14E-7
Organismal survival	3.14E-6–3.14E-6	Tissue development	4.18E-4–4.41E-7
Nervous system development	3.68E-3–3.47E-6	Tissue morphology	3.14E-4–1.34E-6
Tissue development	3.96E-3–3.47E-6	Connective tissue development	3.14E-4–1.34E-6

^a^*p*-Value calculated by Fisher’s Exact test.

### qPCR array analysis of canonical pathways validates IPA prediction of dysregulation

We next set out to confirm the observed dysregulation of some of the identified canonical pathways, particularly those pathways with the most significant changes. Specifically, we used qPCR to analyze the pathways with an activation score of less than −1 (z<−1) and greater than +1 (z>+1) in the 48 hpi dataset. While the NER pathway is the most significant and most downregulated pathway, the closely related cell cycle and cell death pathways have previously been reported to be dysregulated during ZIKV infection [4]. Therefore, we decided to test the expression profile of genes in the “Ephrin Receptor Pathway” (z=−3.464 and –log(p) = 4.68), which is the most significantly downregulated pathway after the NER pathway (Figure 2(c) and Table S3). Among the upregulated pathways, we tested expression profile of genes in the “PPAR signaling” pathway (z=1.134, –log(p) = 2.59), which is the most significant among pathways with activation scores above 1.

**Figure 3.**
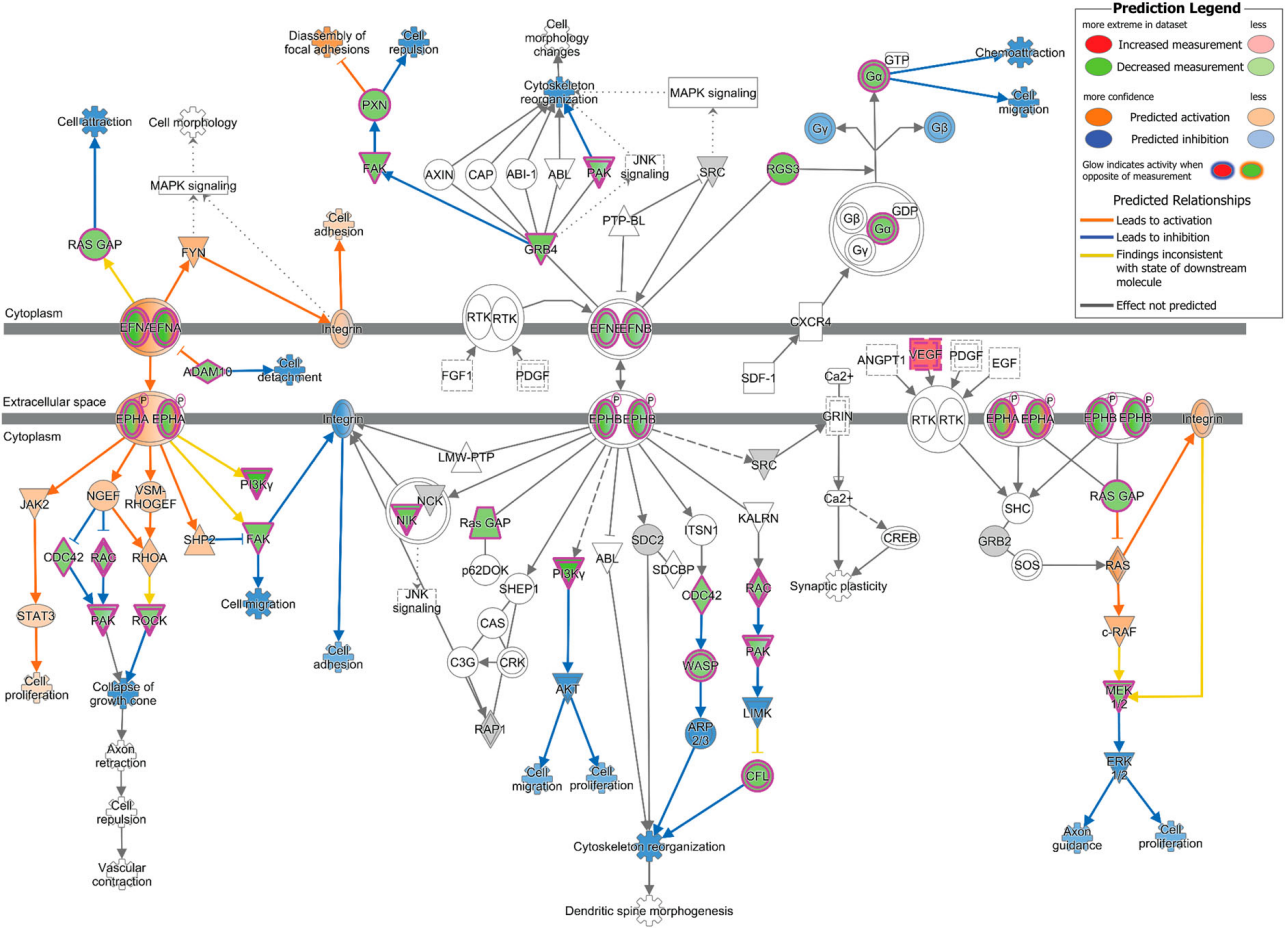
Downstream Effect prediction on Ephrin signaling pathway by IPA. The gene expression data obtained by qPCR array in infected cells was overlaid on Ephrin signaling pathway using IPA. The biological functions affected by the gene expression changes was predicted using the Molecular Activity Predictor (MAP) tool in IPA. Proteins upregulated in the qPCR experiment are represented in shades of red and those that are downregulated are represented in shades of green, where the intensity of the shade is representative of the relative level of upregulation or downregulation. The proteins and downstream functions that are predicted to be activated or inhibited due to the observed gene expression changes are represented in shades of orange and blue respectively, where the intensity of the shade is representative of the relative level of activation or inhibition.

qPCR array of 88 genes belonging to the Ephrin receptor pathway revealed 50 genes to be downregulated by more than 1.2 fold and 6 genes to be upregulated by more than 1.2 fold among all the genes tested (Figure S1). Overlay of the qPCR array data on the Ephrin receptor pathway in IPA demonstrated repression of several downstream effects of the signaling pathway that are related to neuronal development (Figure 3). The observed downregulation of the PAK and ROCK proteins is predicted to affect “collapse of the growth cone,” while downregulation of FAK and CFL proteins inhibits “cell migration” and “cytoskeletal organization.” Downregulation of MEK1/2 is predicted to inhibit Axon guidance and cell proliferation via inhibition of ERK1/2. Moreover, downregulation of other proteins in the Ephrin signaling pathway, such as RASA1 (RAS GAP), PXN and Gα has been shown to affect “cell attraction,” “cell repulsion” and “chemoattraction” respectively.

**Figure 4.**
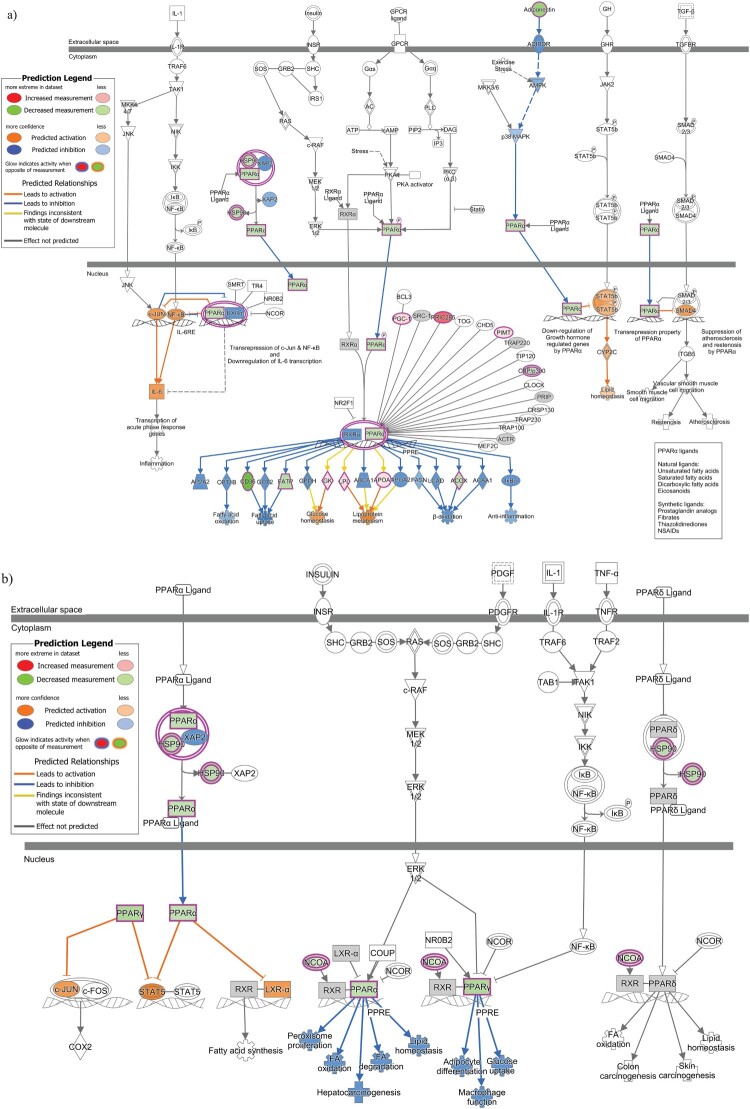
Downstream Effects prediction on PPAR signaling pathways by IPA. (a) The gene expression data observed by qPCR array in infected cells was overlaid on PPAR alpha signaling pathway using IPA. (b) The gene expression data observed by qPCR array in infected cells was overlaid on PPAR signaling pathway using IPA. The biological functions affected by the gene expression changes was predicted using the Molecular Activity Predictor (MAP) tool in IPA. Proteins upregulated in the qPCR experiment are represented in shades of red and those that are downregulated are represented in shades of green, where the intensity of the shade is representative of the relative level of upregulation or downregulation. The proteins and downstream functions that are predicted to be activated or inhibited due to the observed gene expression changes are represented in shades of orange and blue respectively, where the intensity of the shade is representative of the relative level of activation or inhibition.

qPCR array of 86 genes belonging to the PPAR signaling pathway revealed 23 genes to be downregulated and 33 genes to be upregulated by more than 1.2 fold among all the genes tested (Figure S2). The PPAR pathway involves the PPAR family of receptors, which consists of PPARα, PPARγ and PPARδ [28]. An overlay of the PPAR pathway qPCR data on the signaling pathway involving exclusively PPARα and its partners is shown in Figure 4(a). Figure 4(b) on the other hand, is a depiction of the overlay of the PPAR pathway qPCR data on the signaling pathway involving all three PPAR family of receptors. In the PPARα/RXRα activation pathway, the observed upregulation of APOA1 and LPL proteins is predicted to result in dysregulation of lipoprotein metabolism, while upregulation of GK activates glucose homeostasis. The observed downregulation of FATP (SLC27A1), CD36 and ACOX proteins is predicted to repress “Fatty acid uptake” and “β-oxidation” respectively. In PPAR signaling pathway, while the observed downregulation of PPARα is predicted to affect the lipid environment by affecting functions such as Fatty acid degradation, oxidation and lipid homeostasis, downregulation of PPARγ is predicted to affect glucose uptake.

### Immunofluorescence analysis confirms overexpression of genes in the PPAR pathway at the protein level

**Figure 5.**
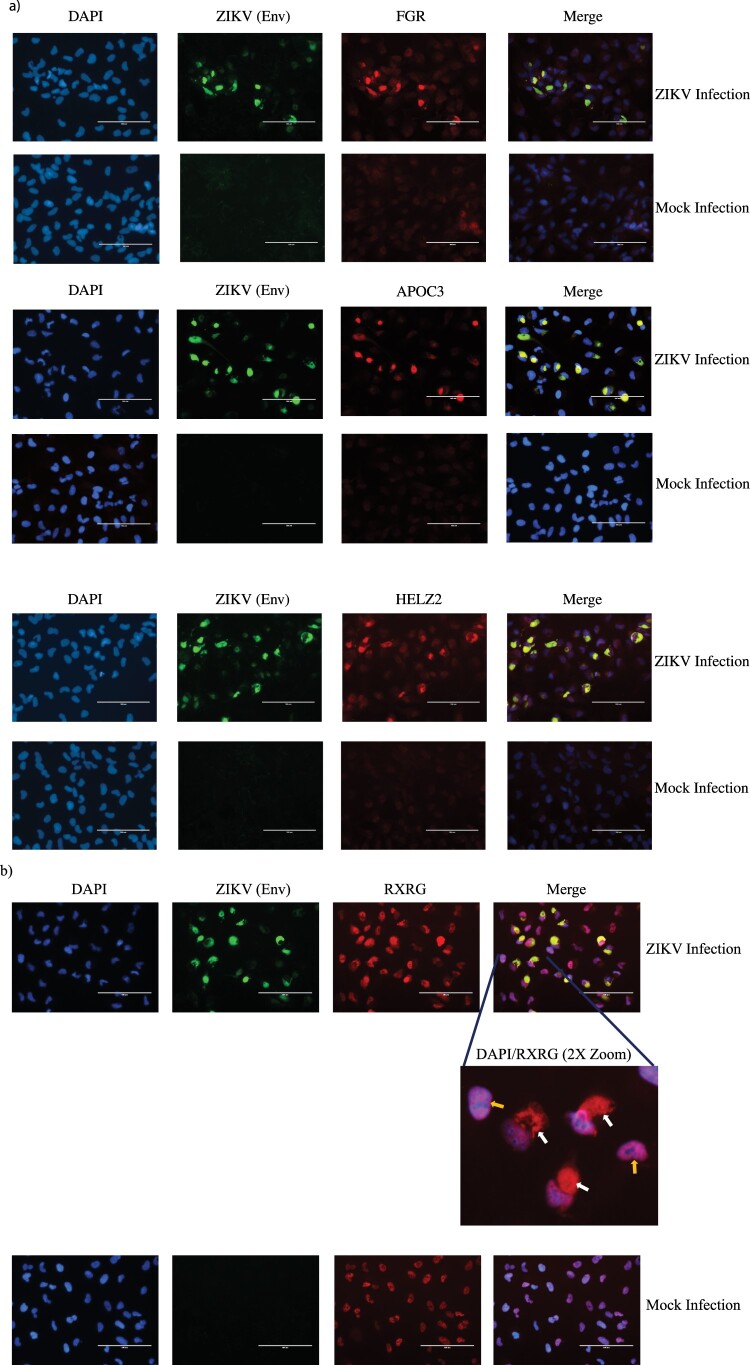
Immunofluorescent staining to detect expression of PPAR signaling pathway genes in infected cells. (a) Protein-level expression of PPAR pathway genes was monitored by immunostaining with antibodies against FGR, APOC3 or HELZ2 (red) in ZIKV-infected and mock-infected ReNcell CX cells. Infected cells were identified by staining with antibody against ZIKV Envelope protein (green) and nuclei were stained using DAPI (blue). Scale bar is 100 µm. (b) Protein-level expression of PPAR pathway gene RXRG was monitored by immunostaining with antibodies against the protein (red) in ZIKV-infected and mock-infected ReNcell CX cells. Infected cells were identified by staining with antibody against ZIKV Envelope protein (green) and nuclei were stained using DAPI (blue). Scale bar is 100 µm. A 2X zoom of a specific region in the infected cell field of view is shown to indicate cytoplasmic staining of RXRG in infected cells. White arrows indicate cytoplasmic staining of the protein in infected cells and yellow arrows indicate RXRG staining limited to the nucleus in mock-infected cells.

Four genes belonging to the PPAR signaling pathway, namely, FGR, APOC3, HELZ2 and RXRG were discovered to be highly upregulated by qPCR (Figure S2). We assessed the protein-level expression profile of these four genes in infected cells using immunofluorescence. Cortical neural progenitor cells are the main cell population affected by ZIKV infection [4]. Therefore, we performed immunofluorescent staining in ZIKV-infected ReNcell CX cells, which is an immortalized cell line derived from human cortical neural progenitor cells that expresses NPC markers (Figure S3) [29]. As shown in Figure 5(a and b), we indeed observed increased staining for the above-mentioned proteins in infected cells compared to mock infected cells, in agreement with the results from the qPCR array data. Interestingly, we also observed altered cellular localization of RXRG protein in ZIKV-infected cells when compared to uninfected cells (Figure 5(b)).

## Discussion

ZIKV infection, like other flavivirus infections, is known to alter expression of host proteins and modulate cellular signaling pathways [6, 7]. While Several “omics” studies have identified various biological functions that are altered during ZIKV infection in the host cell, a complete understanding of the complex virus-host interactions is yet to be articulated [6, 7]. In our study, we primarily sought to investigate the pathways that could drive the changes in biological functions, by analyzing the proteomics dataset using IPA. A summary of previous “omics” studies and how the major findings in those studies compare with our study is presented in Table S6. As summarized in the table, many of the studies concur that ZIKV infection of NPCs leads to major alterations in biological functions related to cell cycle regulation, cell death and neurogenesis. While we observed similar alterations in biological functions in this study, further investigation using IPA led to the identification of deregulation in several cellular signaling pathways including Ephrin and PPAR signaling pathway during ZIKV infection.

Through this analysis, we identified novel host signaling pathways modulated during ZIKV infection of NPCs, which are the primary cell type target of ZIKV [4]. However, as the immunofluorescence data indicated that only a fraction of the cells in the cultures were infected, some of the identified alterations could be derived from uninfected bystander cells stimulated by paracrine signals such as type I IFN (Figure 1(c)). While the interpretation of our results are limited by the low percentage of infected cells, it is worthy mentioning that the protein-level expression changes that we observed by immunofluorescent staining were restricted only to the infected cells and not observed in uninfected cells (Figure 5).

ZIKV is classified into two major lineages or strains, the Asian/American and African. Several studies have shown various differences between the two strains, such as differences in infection rate, viral particle production, cellular and antiviral responses and sensitivity to antiviral immunity [30, 31]. Differences in pathogenicity has also been observed between the various subtypes of the Asian/American lineage [32]. Thus, the cellular responses induced by ZIKV could vary depending on the strain used for infection. Therefore, differential expression in the genes and pathways identified in this study is limited to the American strain PRVABC59, which is the lineage associated with the neurological and congenital abnormalities observed in infants during the recent outbreak in the Americas [12].

Mass spectrometry analysis of ZIKV-infected NPCs collected at 24 hpi and 48 hpi revealed several cellular proteins regulated during ZIKV infection. Examination of this dataset using IPA analysis resulted in the prediction of several cellular pathways. It is worth noting that our data revealed the activation of EIF2 signaling pathway at 24 hpi, which is related to the host translational machinery (Figure 2(b)). ZIKV and other flaviviruses rely on the host translational machinery for translation of viral proteins [33]. Activation of the EIF2 signaling pathway at this time point emphasizes the virus requirement for host machinery to carry out protein synthesis and progeny virus production.

Malformations in the brain and central nervous system of infants and adults caused by ZIKV have been extensively studied since the 2015 Brazil outbreak [4]. While the association of the virus with congenital infections of the central nervous system and neurological complications is well documented, the molecular mechanisms by which ZIKV achieves this is not completely understood [1]. As a result, we were interested in probing our dataset for functions and pathways related to neurogenesis and neuronal physiology. A gene ontology analysis indicated that many of the top differentially expressed proteins at 24 hpi such as GAP43, LLG1, DCLK1, DCX, LRP1 and MAP4K4 are involved in axonogenesis and neuron projection development (data not shown). Supporting some of our observations, Jiang et al., presented upregulation and downregulation respectively of Neuromodulin (GAP43) and Doublecortin (DCX) protein in brains of ZIKV infected mice [16].

Interestingly, significantly more and varied cellular signaling pathways were identified to be differentially regulated at 48 hpi than 24 hpi (Figure 2(c)). This shows that the virus exerts a more dynamic and versatile influence on the cellular machinery at 48 hpi, which is also the time point when peak viral titers were observed (Figure 1(b)). Therefore, we decided to study the cellular pathways predicted to be regulated at 48 hpi more closely. Many studies have demonstrated alterations in the DNA damage response, cell cycle and cell death pathways in ZIKV-infected cells [4, 7]. In agreement with these studies, we identified dysregulation of several cell cycle related pathways such as the “NER” and “Cell cycle G2/M DNA damage checkpoint regulation” pathways at 48 hpi (Figure 2(c)).

IPA analysis of the 48 hpi dataset also revealed enrichment for several pathways that are known to affect nervous system function and development. For example, the two pathways validated by qPCR array in our study, Ephrin receptor signaling and PPAR signaling pathway are well known to affect neurogenesis. To our knowledge, this is the first time genes involved in Ephrin receptor signaling and PPAR signaling pathway have been shown to be differentially regulated upon ZIKV infection. While many of the IPA predictions were consistent with the observed differential regulation of the genes by qPCR, some of the findings were inconsistent with the differential regulation of the genes observed by qPCR (yellow lines in Figures 3 and 4). This could be due to crosstalk of other signaling pathways on the gene, as cellular proteins are not confined to a single cellular pathway and are known to affect and/or be affected by multiple signaling pathways. Additionally, it should be noted that the set of genes assessed in the qPCR array for verification of dysregulation in PPAR signaling and Ephrin signaling pathways (Figures S1 and S2) were different from the set of proteins identified by proteomics as belonging to the two pathways (Table S3). Therefore, while we verified the dysregulation in the pathways, as a whole, using approaches at both protein-level (mass spectrometry) and mRNA level (qPCR), the same could not be done for the individual genes.

Ephrin signaling plays an important role in embryonic and adult neurogenesis [34]. In our study, qPCR analysis on genes involved in Ephrin receptor signaling pathway showed downregulation of a large number of genes in the pathway (Figure S1). As shown in Figure 3, the observed alterations in the expression profile of Ephrin signaling genes are predicted to repress several biological functions involved in neuronal development. Neuronal development in a developing brain requires neuronal migration and formation of synaptic connections to assemble and build functional neural circuits [35]. In this process, neurons extend their axons and navigate through tissues to reach target cells and this requires numerous axon guidance cues and receptors that are expressed on the elongating tip of the axons called the “growth cone” [36]. As the axon steers towards its target cell, it receives extracellular stimuli by interacting with various ligands of the extracellular matrix and these ligand–receptor interactions mediate different types of attractive and repulsive cues [36]. The signalling from these ligands, cell adhesion molecules and receptors are channeled for remodelling of the cytoskeleton, which is the ultimate effector for movement and outgrowth of the axon towards its target [36]. Thus, the predicted dysregulation of different downstream functions of the Ephrin receptor pathway (Figure 3) such as cell migration, axon guidance, collapse of growth cone, cell attraction, cell repulsion, chemoattraction, cell adhesion and cytoskeletal organization could contribute to defects in neuronal development observed during congenital Zika syndrome. Indeed, abnormalities in cytoskeletal organization, axon guidance, growth cone collapse, cell migration, cell attraction, cell repulsion and cell adhesion can result in neurodevelopmental disorders such as autism, lissencephaly, intellectual disabilities, polymicrogyria, microcephaly, epilepsy and schizophrenia [37, 38]. Additionally, considering the known redundancy in entry receptor and attachment factor usage by ZIKV virus [39], it is noteworthy to mention that many Ephrin receptors are known entry receptors for viruses such as KSHV, EBV and HCV [40].

The nuclear receptors PPARs are ligand-activated transcription factors that participate in the regulation of lipid and carbohydrate metabolism [28]. PPARs can affect neurogenesis and neurodegenerative diseases and PPAR-deficient mice have been shown to have defects in the central nervous system [41]. In our study, the observed alterations in expression of genes belonging to the PPAR signaling pathway (Figure S2) is predicted to alter many functions related to lipid and glucose metabolism (Figure 4(a and b)). While NPCs predominantly use glycolytic pathways for energy generation, lipogenic pathways are important for sustaining NPC proliferation and inhibition of this pathway decreases their proliferative capability [42]. Moreover, recent studies show that the cells utilize fatty acid oxidation to maintain neurogenic activity [43]. Differentiation of NPCs into various neuronal lineages is known to be accompanied with alterations in lipid and glucose metabolic pathways and their differentiation potential is defined by their ability to adapt to different metabolic states [43]. In early brain development, NPCs initially divide symmetrically to a sufficient number before differentiating into the neuronal lineages [44]. Thus, NPC proliferation and differentiation is highly regulated and any pathway that affects these two functions could have damaging effects on brain development. Considering the known relationship between metabolic imbalances and neurodegenerative disorders, the necessity for investigating the link between ZIKV-induced altered metabolic pathways, including PPAR signaling pathway and ZIKV-induced microcephaly is warranted. Other signaling pathways identified in our study that could affect nervous system development include BAG2 signaling pathway [45], RhoGDI signaling and signaling by Rho family GTPases [46], GNRH signaling [47] and Endothelin-1 signaling [48].

In our work, we also performed immunofluorescence staining to confirm protein-level overexpression of some of the genes belonging to the PPAR signaling pathway (Figure 5). We tested the expression of the four highly expressed genes in the PPAR qPCR array, namely FGR, APOC3, HELZ2 and RXRG (Figure 5(a and b)). The expression of all the four genes were upregulated in the infected cells in line with the qPCR array results. These four proteins have different roles in the PPAR signaling pathway. FGR is a non-receptor tyrosine protein kinase, which belongs to the Src family of tyrosine protein kinases [49]. Src kinases such as FGR act as cofactors in PPAR signaling pathway [50]. APOC3 protein functions in lipid transport and is an important regulator of triglyceride metabolism [51]. HELZ2 is a co-activator of the PPAR receptors and affects the transcriptional regulation of their targets [52]. RXRG protein can bind PPAR receptors and regulate transcription of target genes as a RXR/PPAR heterodimeric protein [28]. While these proteins have important function in PPAR signaling pathway, some of them are also known to impact flavivirus life cycle. For example, HELZ2 helps infected cells establish an antiviral intracellular lipid state that is detrimental for dengue virus life cycle [53]. APOC3 is known to associate with HCV viral particles among various other apolipoproteins and exogenous APOC3 expression is able to rescue low infectious particle production caused due to knockout of ApoE and ApoB proteins in HCV infected cells [54]. While FGR kinase has not been reported to affect flavivirus replication, the related c-Src and c-Yes kinase have been shown to play a role in Dengue and West Nile virus life cycle. RXRG has no known function in virus infections. RXRG is a nuclear receptor and therefore, in our study, it is not surprising to observe nuclear localization of the protein in uninfected cells (Figure 5(b)). However, it is interesting that RXRG exhibits cytoplasmic localization in addition to nuclear localization in ZIKV-infected cells. RXR proteins have diverse nuclear receptor partners and can translocate to the cytoplasm from the nucleus by associating with some of its partners [55, 56]. Such cytoplasmic translocations results in a gain or loss of function for either the RXR receptor or its partner. Therefore, while the precise role and the reason for RXRG cytoplasmic localization in ZIKV-infected cells are unknown, the nuclear export of the protein could be driven by a change in the association of its binding partner in the infected cells. Notably, all the four proteins analyzed by immunofluorescence staining, including the nuclear transcription factors HELZ2 and RXRG localize in the cytoplasm of infected cells (Figure 5). This is not surprising, as virus infections are known to perturb the spatio-temporal organization of cellular proteins and flaviviruses recruit nuclear proteins to the cytoplasm to aid in viral replication and evade host immune sensing [57, 58]. Nonetheless, the overlapping localization pattern FGR, APOC3, HELZ2 and RXRG with the ZIKV Envelope protein and the known cytoplasmic nature of ZIKV life cycle [59], makes it tempting to speculate that these proteins could have important roles in modulating ZIKV life cycle. However, further studies are warranted in order to conclusively determine the reasons for the close localization of the host proteins with the virus antigen and to understand their role in ZIKV life cycle. Therefore, it is critical to further investigate such host factors to fully understand their role in ZIKV life cycle.

Overall, the observations made in this study, in addition to the alterations in Ephrin Receptor and PPAR signaling pathways have provided a holistic insight into NPC response to ZIKV infection. Future studies, including selected repression or activation of key genes identified in this study could help determine exactly how the altered pathways impact virus-induced pathogenesis and virus replication, which is currently ongoing in our laboratories. It could be envisaged that further dissection of the molecular and cellular pathways could inform the rationale design of future therapeutic intervention.

## Supplementary Material

Table_S6_revised.xlsx

Table_S5.xls

Table_S4.xlsx

Table_S3_2nd_revision.xls

Table_S2_revised_.xlsx

Table_S1_revised.xlsx

Supplementory_Info-revised_FInal_-_track_change_turned_off.docx
