# Supplementary material for: Dysregulation of Ephrin receptor and PPAR signaling pathways in neural progenitor cells infected by Zika virus
Source: Emerg Microbes Infect. 2020 Sep 20;9(1):2046–60. doi: 10.1080/22221751.2020.1818631 (PMC7534353; doi:10.1080/22221751.2020.1818631)
Supplement: Supplementory_Info-revised_FInal_-_track_change_turned_off.docx [file TEMI_A_1818631_SM7166.docx]

**Supplementary Information**

Dysregulation of Ephrin Receptor and PPAR signaling pathways in Neural Progenitor Cells Infected by Zika Virus

Sathya N. Thulasi Raman^1^, Elyse Latreille^1^, Jun Gao^1^, Wanyue Zhang^1,6^, Jianguo Wu^1^, Marsha S. Russell^1^, Lisa Walrond^1^, Terry Cyr^1^, Jessie R. Lavoie^1^, David Safronetz^2^, Jingxin Cao^2^, Simon Sauve^1^, Aaron Farnsworth^1^, Wangxue Chen^3^, Pei-Yong Shi^4^, Youchun Wang^5^, Lisheng Wang^6^, Michael Rosu-Myles^1,6^, Xuguang Li^1,6,*^

**Supplementary materials and methods**

***iPSC cell line culture and maintenance***

Induced pluripotent stem cell (WLS3C-iPSC) were maintained on diluted Matrigel (BD Biosciences, Cat No. 354230) in Essential 8 (E8) media: DMEM/F-12 (Life Technologies, Cat No. 11330), 0.064µg/ml Ascorbic Acid 2 Phosphate Mg (Sigma, Cat No. A8960), 14µg/ml sodium selenium (Sigma, Cat No. S526), 0.1µg/ml FGF2 (Life Technologies, Cat No. PHG0263), 19.4µg/ml human Insulin (Wisent, Cat No. 511-016-CM), 10.7µg/ml transferrin (Sigma, Cat No. T0665), 0.002µg/ml TGF-β1 (Life Technologies, Cat No. PHG9202), 543µg/ml NaHCO3 (Sigma, Cat No. 5761). Media was replaced daily, and iPSCs were passaged every 4-5 days using 0.5mM EDTA. iPSC-derived NPCs were maintained on Matrigel (Corning Life Sciences, Cat. No. 354230) coated flasks in DMEM-F12: Neural Basal medium (1:1) (GIBCO) supplemented with N2 and B27 supplement (GIBCO) at a final concentration of 0.5X, 5 ug/ml human insulin (Wisent), 20 ng/ml each of human FGF2 and EGF (GIBCO), 0.5X Glutamax (GIBCO) and 50 ug/ml Gentamicin (GIBCO).

***iPSC differentiation into Monolayer NPCs***

iPSCs were differentiated into Monolayer NPCs using a modified Chambers protocol [1] as described below. iPSCs were passaged as intact colonies, maintained on Matrigel, and neural lineage differentiation was initiated when the cultures were 90%+ confluent to promote neuroepithelial induction [2], typically 3-5 days post-passage. Neuroectoderm differentiation was initiated by replacing E8 with KSR media containing 10µM SB (SIGMA, Cat No. S4317) and 500nM LDN (Tocris, Cat No. 6053). KSR media (for 500mL) contains 414mL of Knockout-DMEM (Life Tech, Cat No. 10829-018) with 15% Knockout-serum replacement (Life Tech, Cat No. 10828-028), 5mL of 100X Glutamax (Thermo Fisher, Cat No. 35050061), 5mL of 100X MEM-NEAA (Gibco, Cat No. 11140-050), 500uL ß-ME(Thermo Fisher, Cat No. 21985023), and 500µL Gentamicin (Wisent, Cat No. 450-135). Media was changed daily as KSR+ SB & LDN from days 0-3. At day 4, N2 media was added in increasing amounts in the following ratios: days 0-3 100% KSR, days 4-5 75:25 KSR:N2, days 6-7 50:50 KSR:N2, days 8-9 25:75 KSR:N2, days 10-11 100% N2, harvest at day 12. LDN supplementation was maintained at all days, while SB was included only on days 0-3. N2 media composition is as follows (for 500mL): 486.5mL of DMEM-F12, 15% glucose, 5mL N2 supplement, 20µg/mL human insulin, 2.5mL Hepes (Life Tech, Cat No. 15630080), and 500µL Gentamicin. Cells were maintained at 37°C, 5% O_2_, 10% CO_2_ during differentiation.

## ***Proteomics Analysis***

### Materials

Dithiothreitol (DTT, 43815), ammonium bicarbonate (ABC, 09830), iodoacetamide (IOA, I1149), formic acid (FA, 5.33002), LC-MS grade water (WX0001) and LC-MS grade acetonitrile (AX0156) were purchased from Sigma-Aldrich (Oakville, ON, Canada). Sequencing grade trypsin (V5111) and recombinant Lys-C (rLys-C, V1671) were purchased from Promega. Sartorius^TM^ Vivacon^TM^ 500 10 000 MWCO filters (VN01H02) were purchased from Fisher Scientific. PBS was purchased from Thermo Fisher Scientific (14190-144).

### Sample preparation for proteomics

At 24 hpi and 48 hpi, the cells were rinsed with PBS and then the monolayer was scraped and collected in PBS. The cells were spun down and lysed in RIPA (20 mM Tris-Hcl pH 7.5. 150 mM NaCl, 0.5% SDS, 0.5% Triton X-100, 0.5% Na deoxycholate, 1 mM EDTA) with Protease/Phosphatase inhibitor (Thermo Scientific, Cat No. A32959) and sonicated using a Branson Sonifier 150. The proteins in the sample were then precipitated using four times the sample volume of cold acetone. Samples from five biological replicates at each time point were prepared and analyzed by Mass spectrometry for proteomic analysis.

### Reduction, alkylation and trypsin digestion

Acetone-precipitated samples were diluted with 200 µL ABC. Reduction was achieved with the addition of DTT (0.01 M) followed by 30 min incubation at 56 °C. Carbamidomethylation was accomplished with the addition of IOA (0.02 M) followed by incubation for 30 min at RT in the dark. Excess IOA was quenched with DTT prior to buffer exchange. Reduced and alkylated samples were buffer exchanged to 0.05M ABC for protein digestion using Vivacon^TM^ 500, 10 000 MWCO filters. Protein digestion was accomplished using a FASP based approach using rLys-C and trypsin enzymes. First, 100 µL of 0.01 μg/μl rLys-C in 0.05 M ABC was added and the filtrate was collected into fresh tubes at 8000 g over 20 mins of centrifugation. Then, 150 μl of 0.02 μg/μl trypsin was added to the top of the filter and the centrifugation step was repeated. Finally, 100 µL of 0.05 M ABC was added and the sample was centrifuged for 20 mins at 10,000 g. All centrifugation steps were carried out at 37 °C. Fractions were combined and lyophilized overnight. Samples were reconstituted in a sufficient volume of 0.1% FA to yield concentrations ranging from 400-600 ng/µL.

### Reversed-phase liquid chromatography and mass spectrometry

Reversed-phase separation of protein digests was carried out on an Easy nLC 1000 system (ThermoFisher Scientific) equipped with a Thermo Acclaim PepMap® 100 C18 trap column (75 μm x 2cm with 3 μm particles) and a Thermo PepMap RSLC 100 C18 reversed phase analytical column (75 μm x 15 cm with 3 μm particles). Solvent A consisted of 0.1% aqueous FA and solvent B consisted of 0.1% FA in acetonitrile. The temperature was maintained at 30 °C and the flow rate was 0.3 mL/min. The gradient was as follows: initial, 2% B; increasing to 20% B in 40 mins, then 40 % B by 60 mins, and 60% B at 70 mins, increasing to 95% B by 75 mins and held at 95% B for 10 mins before being returned to 2% B for column equilibration. 200-300 ng of reduced and alkylated protein digests were injected on column.

The eluting peptides were analyzed with an Orbitrap^TM^ Fusion^TM^ Tribrid^TM^ mass spectrometer (ThermoFisher Scientific) operating with a 3 s cycle time in DDA ‘Top-Speed’ mode. An EasySpray source was used for ionization in positive ion and standard pressure modes. For MS1, spectra were detected in the Orbitrap using a resolution of 120 000 and an AGC target value of 2E5 and a maximum injection time of 50 ms. MS2 spectra were detected in the ion trap using higher-energy collisional dissociation (HCD) fragmentation with a collision energy set to 30%, an AGC target of 1E4 and a maximum injection time of 35 ms.

### Data Processing

MS/MS spectra were processed using Proteome Discoverer 2.2. A Sequest search was carried out against all Homo sapiens entries in the Swissprot database to identify host proteins present in the samples. A Sequest search was also carried out to identify Zika virus proteins in infected cells and common contaminants encountered during sample processing of Mass spec samples using a custom ZIKV protein database and a contaminants database (cRAP database) respectively. Searches were performed specifying trypsin as the proteolytic enzyme with the following set search criteria: 2 missed cleavages were allowed, carbamidomethylation of cysteine as a fixed modification and N-terminal acetylation as a variable modification. Peptide and fragment ion mass tolerances were set at 6 ppm and 0.6 Da, respectively. Precursor ions were quantified based on peak area abundance. Normalization mode was set to Total Peptide Amount and scaling mode was set to none. Percolator FDR validation was based on q-value using a high confidence threshold of 0.01 and a medium confidence threshold of 0.05.

## ***Bioinformatics and Statistical analysis***

Gene Ontology Enrichment analysis was performed using Panther Overrepresentation test with GO Ontology database released on 2019-12-09. Statistical analysis was performed using Fisher Exact’s test with FDR correction.

Proteomics and qPCR array data were imported into IPA and analyzed using the same (Qiagen, Content version – 47547484, Release Date – 2019-02-08). The Canonical pathways identified by IPA , their predicted activation states and downstream effects are based on the curated literature database in IPA. The significance of the association between the data set and the canonical pathway is determined by two parameters: (1) A ratio of the number of proteins from the data set that map to the pathway divided by the total number of proteins that map to the canonical pathway in IPA knowledgebase and (2) a p-value calculated using Fischer's exact test determining the probability that the association between the proteins in the data set and the canonical pathway is due to chance alone. The p-value (“enrichment” score) assesses which biological attributes are significantly associated with the dataset. A –log(p-value) ≥1.3 was considered significant. The p value for a given disease or biological function is calculated by IPA software after taking into account the total number of proteins in the dataset that participate in that process in relation to the total number of proteins linked with that process in IPA knowledgebase. The z-score mentioned in Fig. 2b and 2c for canonical pathways is a measure of expected correlation between the direction of the overall functional endpoints of a pathway and the direction of differentially expressed genes in the pathway. Therefore, canonical pathways with a z-score above zero are predicted to be activated, while those with a z-score below zero are predicted to be inhibited with respect to their functional endpoints. Figure 2b and 2c were generated in GraphPad Prism software (Version 7, Release date – March 31, 2016) using the z-score and –log(p) value data for Canonical pathways generated by IPA.

Proteomics data from five biological replicates were used for statistical analysis. Only those proteins which were identified in at least 3 out of the 5 replicates and with 95% or higher confidence at the peptide and protein levels were considered for further analyses. Missing values are imputed with the average values of the arm (either Infected or Control) of their corresponding proteins. The primary differential expression analysis (Infected vs. Control) was carried out using a two-sample t-test, and the results from the primary analysis were further confirmed with those of a non-parametric approach (Wilcoxon rank sum test), which suggests the potential impacts of non-normality on the primary analysis are limited. The p-values of the primary analysis were adjusted for multiplicity by a false discovery rate (FDR) controlling method based on the linear step-up method suggested by Benjamini and Hochberg [3]. The significance of differential expression of a protein was determined by using an alpha level of 0.05 of FDR and the fold change threshold of ≥+1.2-fold or ≤-1.2 fold (vs. Control). The statistical analyses were implemented by using SAS Enterprise Guide 5.1/7.1.

**References**

1. Chambers SM, Fasano CA, Papapetrou EP, Tomishima M, Sadelain M, Studer L. Highly efficient neural conversion of human ES and iPS cells by dual inhibition of SMAD signaling. Nat Biotechnol. 2009 Mar;27(3):275-80.

2. Tchieu J, Zimmer B, Fattahi F, Amin S, Zeltner N, Chen S, et al. A Modular Platform for Differentiation of Human PSCs into All Major Ectodermal Lineages. Cell Stem Cell. 2017 Sep 7;21(3):399,410.e7.

3. Benjamini Y, Hochberg Y. Controlling the False Discovery Rate: A Practical and Powerful Approach to Multiple Testing. Journal of the Royal Statistical Society.Series B (Methodological). 1995 2020/03;57(1):289-300.

**Supplementary Figure Legends**

Figure S1. Ephrin receptor pathway gene expression changes analyzed by qPCR array. The changes in the mRNA level of genes belonging to Ephrin receptor signaling pathway were determined using a qPCR array. The mRNA level of the genes were normalized to GAPDH mRNA levels and fold change in gene expression was calculated by comparing to gene expression levels in mock-infected cells (control). The data was used to plot a graph using GraphPad Prism (Version 7) and the error bars represent standard deviation among replicates.

Figure S2. PPAR pathway gene expression changes analyzed by qPCR array. The changes in the mRNA level of genes belonging to PPAR signaling pathway were determined using a qPCR array. The mRNA level of the genes were normalized to GAPDH mRNA levels and fold change in gene expression was calculated by comparing to gene expression levels in mock-infected cells (control). The data was used to plot a graph using GraphPad Prism (Version 7) and the error bars represent standard deviation among replicates.

Figure S3. Immunofluorescent staining of ReNcell CX cells with antibodies against NPC markers. ReNcell CX cells were immunostained with primary (1^◦^) antibodies against NPC markers SOX2 (red) and Nestin (green) (Top panel) or Pax6 (red) (bottom panel). As a negative control, NPC cells were immunostained with secondary (2^◦^ ) antibodies alone. The nuclei were visualized using DAPI (blue). Scale bar is 200 µm.
